# Characterization and Evaluation of Neuronal Trans-Differentiation with Electrophysiological Properties of Mesenchymal Stem Cells Isolated from Porcine Endometrium

**DOI:** 10.3390/ijms160510934

**Published:** 2015-05-14

**Authors:** Raghavendra Baregundi Subbarao, Imran Ullah, Eun-Jin Kim, Si-Jung Jang, Won-Jae Lee, Ryoung Hoon Jeon, Dawon Kang, Sung-Lim Lee, Bong-Wook Park, Gyu-Jin Rho

**Affiliations:** 1 Department of Theriogenology and Biotechnology, College of Veterinary Medicine, Gyeongsang National University, Jinju 660-701, Korea; E-Mails: raghu_dvs@yahoo.com (R.B.S.); imran.bch@gmail.com (I.U.); sjjang@gnu.ac.kr (S.-J.J.); iamcyshd@nate.com (W.-J.L.); rhjeon@gnu.ac.kr (R.H.J.); sllee@gnu.ac.kr (S.-L.L.); 2 Department of Physiology and Institute of Health Sciences, School of Medicine, Gyeongsang National University, Jinju 660-751, Korea; E-Mails: eunjin1981@hanmail.net (E.-J.K.); dawon@gnu.ac.kr (D.K.); 3 Department of Oral and Maxillofacial Surgery, Institute of Health Science, School of Medicine, Gyeongsang National University, Jinju 660-751, Korea; E-Mail: parkbw@gnu.ac.kr; 4 Research Institute of Life Sciences, Gyeongsang National University, Jinju 660-701, Korea

**Keywords:** porcine, endometrium, mesenchymal stem cells, multilineage differentiation, transdifferentiation

## Abstract

Endometrial stromal cells (EMSCs) obtained from porcine uterus (*n* = 6) were positive for mesenchymal stem cell markers (CD29, CD44 and CD90), and negative for epithelial marker CD9 and hematopoietic markers CD34, CD45 analyzed by flow cytometry. Further the cells were positive for expression of mesenchymal markers, *CD105*, *CD140b*, and *CD144* by PCR. Pluripotent markers OCT4, SOX2, and NANOG were positively expressed in EMSCs analyzed by Western blotting and PCR. Further, differentiation into adipocytes and osteocytes was confirmed by cytochemical staining and lineage specific gene expression by quantitative realtime-PCR. Adipocyte (*FABP*, *LPL*, *AP2*) and osteocyte specific genes (*ON*, *BG*, *RUNX2*) in differentiated EMSCs showed significant (*p* < 0.05) increase in expression compared to undifferentiated control cells. Neurogenic transdifferentiation of EMSCs exhibited distinctive dendritic morphology with axon projections and neuronal specific genes, *NFM*, *NGF*, *MBP*, *NES*, *B3T* and *MAP2* and proteins, B3T, NFM, NGF, and TRKA were positively expressed in neuronal differentiated cells. Functional analysis of neuronal differentiated EMSCs displayed voltage-dependence and kinetics for transient outward K^+^ currents (*I*_to_), at holding potential of −80 mV, Na^+^ currents and during current clamp, neuronal differentiated EMSCs was more negative than that of control EMSCs. Porcine EMSCs is a suitable model for studying molecular mechanism of transdifferentiation, assessment of electrophysiological properties and their efficiency during *in vivo* transplantation.

## 1. Introduction

Porcine models are of considerable interest in biomedical research due to their similarities with humans in morphology, physiology and developmental characteristics, which makes them a potential donor for organ transplantation and cell therapy. Mesenchymal stem cells (MSCs) have been isolated from various tissues [1,2,3,4] mainly for their potential application in regenerative therapy. Despite that bone marrow aspiration is an invasive procedure, bone marrow derived MSCs are the most commonly studied MSCs for their strong stem cell characteristics [5]. MSCs are small populations of cells that lacks the complete pluripotency unlike embryonic stem cells but have the potential to regenerate complete tissue from which they originate [6].

The porcine endometrium undergoes cycles of growth and apoptosis rather than physical shedding of surface endometrium like in menstruating animals [7]. The endometrium is a highly regenerative tissue divided into two layers, upper functionalis containing epithelial cells and basalis consisting of stromal cells as major cells apart from endothelial cells, leukocytes and blood cells [8]. The basalis region in human consists of adult stromal stem cells which are similar to bone marrow MSCs with strong ability to differentiate into chondrocytes, adipocytes osteocytes [9,10], neuronal cells [11], cardiomyocytes [12], and pancreatic islet cells [13]. It was previously reported that the endometrial stromal region in porcine possesses MSCs [7,14], however complete characterization and transdifferentiation potential of porcine endometrium derived MSCs have not yet been reported. Nuclear reprogramming and changes in transcription of key developmental genes mediate transdifferentiation [15]. Though the MSCs have capacity for mesenchymal lineage differentiation, their transdifferentiation ability needs to be evaluated [16], hence *in vitro* differentiation and transdifferentiation should be assessed at the molecular level in porcine models. However no reports on neuronal trans-differentiation potential of porcine endometrium stromal derived MSCs (EMSCs) has been reported earlier. Most of neuronal induction protocols employ a combination of growth factors and chemical agents [5,17,18,19]. The MSCs neuronal differentiation widely used Woodbury [17] protocol, consisting of β-mercaptoethanol, dimethyl sulfoxide and butylated hydroxyanizole [5,20], failed to exhibit voltage potentials that are a functional characteristic of neurons [21]. Despite the facts that chemical induction causes harmful effects, they are widely employed in stem cell/MSCs differentiation studies [4]. In addition to the chemical inducers, growth factors with trans-retinoic acid (RA), a vitamin A derivative, was found to initiate the neuronal phenotype [5,19,22]. However, there are no reports of studies conducted to assess electrophysiological properties of neuronal transdifferentiated cells from porcine MSCs [5] using RA in combination with growth factors.

In the present study, we characterized the mesenchymal stem cells isolated from porcine endometrial stromal layer for their cluster of determination (CD) markers, pluripotency markers, multilineage differentiation into adipocytes and osteocytes and their trans-differentiation capacity to neuron-like cells. Finally differentiated neurons were subjected to electrophysiological assessment to confirm their intrinsic neuronal functionality.

## 2. Results

### 2.1. Morphology, Cell Surface Markers and Pluripotent Markers

**Figure 1:**
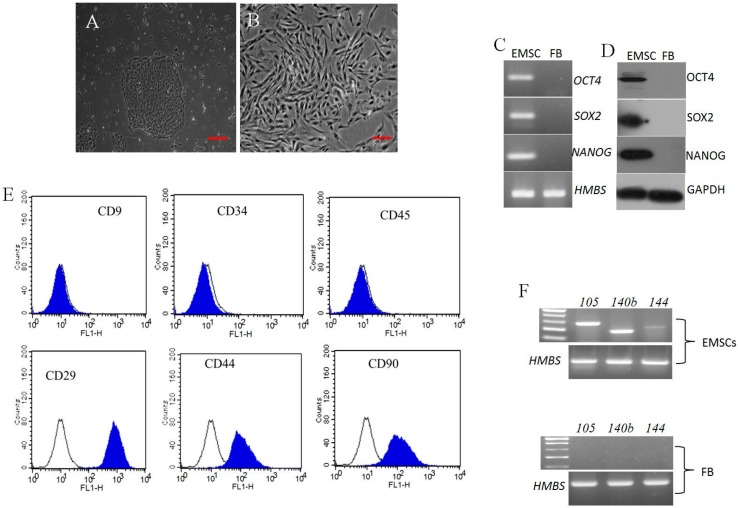

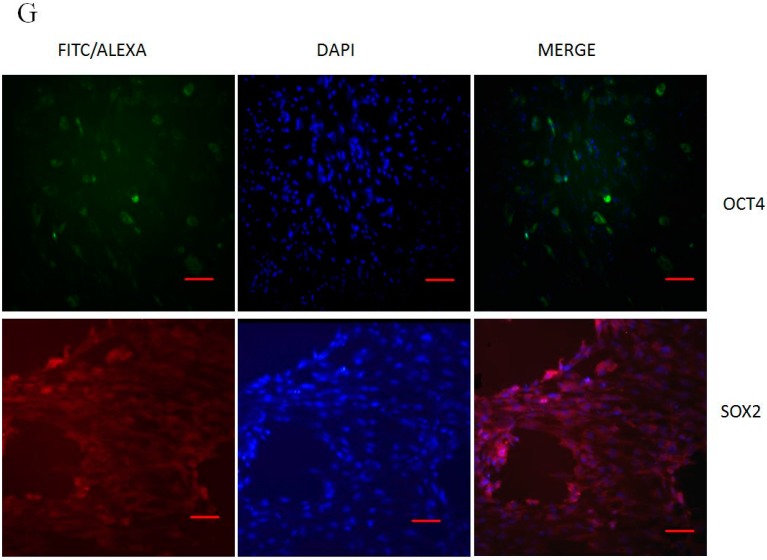
Characterization of porcine endometrial stromal cells (**A**) Single cell colony (Scale bar = 50 μm); (**B**) At 14 days of culture, colony displaying fibroblast like morphology (Scale bar = 100 μm). Pluripotent gene expression analysis in EMSCs and porcine fibroblast cells; (**C**) PCR product after gel electrophoresis, *HMBS* as internal control gene; (**D**) Western blot showing positive expression of OCT4, SOX2 and NANOG. GADPH was used as an internal control; (**E**) CD markers analysis by flow cytometry; Color filled histogram represents specific surface maker and open histograms refers to isotype controls. EMSCs were strongly positive (>94%) for CD29, CD44, CD90, and negative (<2%) for CD34, CD45, and epithelial surface marker CD9; (**F**) PCR analysis of cell surface markers *CD105*, *CD140b* and *CD144* from passage 3 EMSCs and fibroblast as negative control by PCR. *HBMS* was used as an internal control; (**G**) Immunofluorescence analysis of passage 3 EMSCs showing positive expression of OCT4 and SOX2 pluripotent markers (Scale bar = 100 μm).

During primary culture, porcine endometrial stromal cells plated at a cell density of 500 cells/cm^3^ displayed both small and large colonies with densely packed cells. Cells picked from large colonies upon sub-culturing at passage 3 were homogenous and exhibited uniform fibroblast-like morphology (Figure 1A,B). The isolated cells were positive for mesenchymal cell surface makers CD29, CD44, and CD90 (100 ± 0.68, 95 ± 0.54, and 94 ± 0.61, respectively) confirmed by flow cytometry (Figure 1E). However CD9 (0.9 ± 0.28) an epithelial cell surface marker, CD34 and CD45 (2 ± 0.59, 1 ± 0.42) hematopoietic stem cell markers were found to be negative. Further, *CD105*, *CD140b*, *CD144* mesenchymal markers analyzed by RT-PCR were found expressed in passage 3 EMSCs and were absent in ear skin fibroblast cells (Figure 1F).

Pluripotent markers OCT4, SOX2, and NANOG were positively expressed in EMSCs analyzed by western blotting (Figure 1D), semi-Quantitative PCR (Figure 1C), but were not expressed in porcine ear skin fibroblast cells. Following immunostaining for pluripotent markers, OCT4 was moderately expressed, however SOX2 was strongly expressed in passage 3 EMSCs (Figure 1G).

### 2.2. In Vitro Differentiation into Adipocytes and Osteocytes

**Figure 2:**
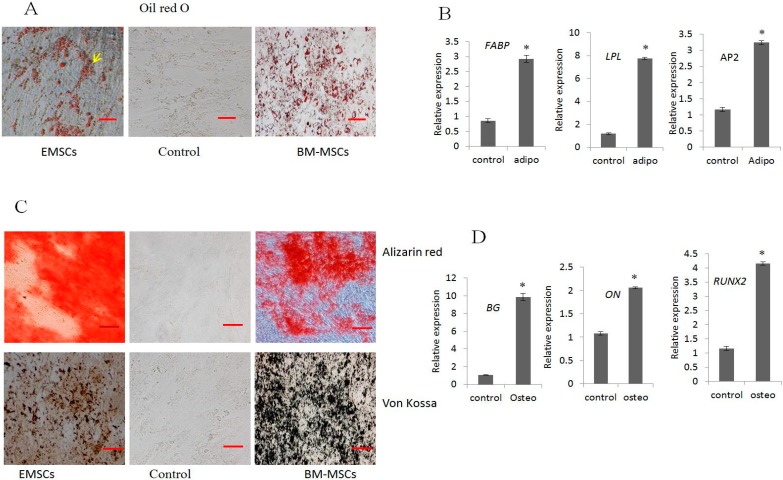
Mesenchymal differentiation potential of EMSCs to adipocytes and osteocytes compared to BMSCs. (**A**) Oil red O staining of lipid droplets; (**B**) RT-qPCR expression of adipocyte specific genes; (**C**) Von Kossa, Alizarin Red staining of mineralization of calcium deposit from differentiated cells; and (**D**) Osteogenic specific gene expression by RT-qPCR. HMBS was used for normalization. Scale bar = 50 μm. (* indicates significant differences (*p* < 0.05) in expression of mRNA between differentiated and untreated control EMSCs).

EMSCs and BM-MSCs (positive control) under specific media condition were induced to differentiate into adipocytes and osteocytes and stained for cytochemical changes. Adipogenic differentiation revealed lipid droplets which were confirmed by Oil Red O staining (Figure 2A). The real time PCR analysis of adipocyte specific gene expressions such as fatty acid binding protein (*FABP*), lipoprotein lipase (*LPL*), and adipocyte protein (*AP2*) (Figure 2B) were significantly (*p* < 0.05) highly expressed in differentiated cells compared to control cells.

Osteogenic differentiation was evidenced by changes in phenotype similar to osteocyte at day 21, and determined by cytochemical staining of calcium deposit and extracellular mineralization matrix by Alizarin Red and Von Kossa (Figure 2C). The expression of osteocyte specific genes such as, osteonectin (*ON*), runt-related transcription factor2 (*RUNX2*), biglycan (*BG*) by RT-PCR showed significant (*p* < 0.05) increase in expression compared to undifferentiated control cells (Figure 2D).

### 2.3. Neurogenic Differentiation

**Figure 3:**
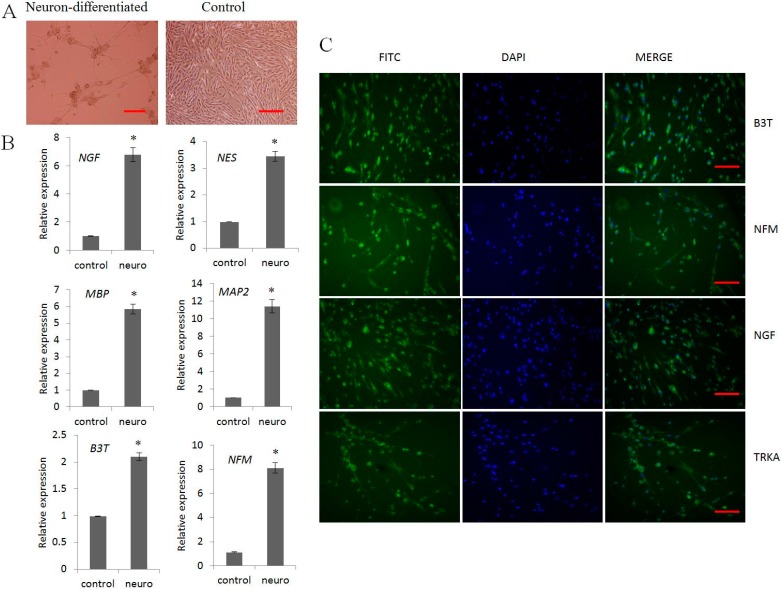
*In vitro* differentiation of porcine EMSCs into neuron-like cells. (**A**) Morphological changes after two days of induction compared to untreated control cells; (**B**) RT-qPCR analysis of neurogenic specific transcripts *NGF*, *NES*, *MBP*, *MAP2*, *B3T*, *NFM* showed significantly higher expression in induced cells compared to non-induced EMSCs control cells; and (**C**) Immunofluorescence analysis showed positive expression of B3T, NFM, NGF, and TRKA in neuronal induced cells which were absent in non-induced control cells. DAPI indicates the nucleus and MERGE indicates the positive expression of protein, FITC conjugated IgG secondary antibody. Scale bar = 100 μm. (* indicates significant differences (*p* < 0.05) in expression of mRNA between neuronal differentiated cells and untreated control EMSCs.). HMBS was used for normalization.

Upon neuronal transdifferentiation, EMSCs exhibited typical neuron-like morphological changes including long axon projections (Figure 3A). At pre-induction during 24 h, the morphological changes were not prominent as post induction with RA progressed; the cells acquired neuronal phenotype which at day 15 was more distinct. The expression of neural markers was strongly positive both at protein and gene levels, evidenced by immunostaining and RT-qPCR. Neuron specific genes, such as *B3T*, *NF*, *NGF*, *NES*, *MBP*, and *MAP2* were significantly (*p* < 0.05) up-regulated in differentiated cells compared to untreated control (Figure 3B).

Immunostaining with neuronal specific antibodies, such as B3T, NFM, NGF, and TRKA, were positively expressed (Figure 3C) in differentiated cells, which were not detected in undifferentiated control cells.

### 2.4. Functional Analysis of Differentiated EMSCs into Neurogenic Cells by Electrophysiology

**Figure 4:**
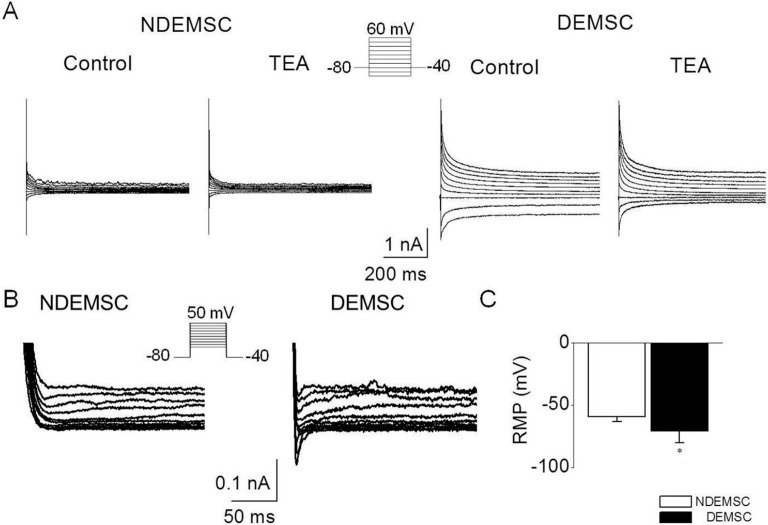
Electrophysiological analysis of porcine EMSCs and neuronal DEMSCs. (**A**) Comparison of K^+^ currents between undifferentiated EMSC (NDEMSC) and differentiated into neurogenic cells (DEMSC). Membrane currents were recorded with the 600-ms voltage steps as shown in the inset in the absence and presence of 3 mM TEA (*n* = 30); (**B**) Representative traces of Na^+^ currents obtained in NDEMSC and DEMSC. Voltage-dependent Na^+^ currents were recorded in DEMSC in response to 100-ms voltage steps (*n* = 30); (**C**) Comparison of resting membrane potential between NDEMSC and DEMSC. Each bar represents the mean ± SD. The (*) indicates a significant difference from the NDEMSC (*p* < 0.05).

We performed the whole cell patch clamp recording to detect the presence of active K^+^ and Na^+^ currents in neuronal differentiated EMSCs. The presence of outward currents (K^+^) were recorded using K^+^-based pipette solution in both DEMSCs and control EMSCs, but the currents showed voltage-dependence and kinetics for transient outward K^+^ currents (*I*_to_) in neuronal differentiated EMSCs. The voltage steps applied from a holding potential of −80 mV to various potentials (−120 to +60 mV), elicited large currents in DEMSCs compared to controls (Figure 4A). The currents were partially inhibited by treatment with 3 mM TEA (approximately 30% ± 15%), indicating that *I*_to_ are present only in DEMSCs and not in control EMSCs. Na^+^ currents were also recorded in neuronal differentiated cells, but not in control EMSCs, in response to voltage steps (Figure 4B), however addition of sodium channel inhibitors, lidocaine (100 μM) and bupivacaine (100 μM) completely blocked the sodium currents (data not shown). Under current clamp conditions, the resting membrane potential of differentiated EMSCs was more negative than that of controls (*n* = 30) (−62 ± 6 mV in DEMSCs *vs.* −50 ± 5 mV in control, Figure 4C). These results showed that voltage-dependent K^+^ and Na^+^ channels are functionally expressed in DEMSCs though the amplitude was low.

## 3. Discussion

In the present study we successfully isolated the porcine endometrial stromal derived mesenchymal stem cells (EMSCs) by plating 500 cells/cm^3^ on culture dish, utilizing the plastic adhering property of MSCs. MSCs are defined as plastic adhering cells, displaying characteristic surface markers along with a potential to differentiate into adipocytes, osteocytes and chondrocytes *in vitro* [23]. The mucosal lining of human endometrium is considered to be highly regenerative tissue and harbors stem/progenitor cells [8,10]. There was a hypothesis that, endometrium regeneration is mediated by stem cells from the basalis region, but not from the functionalis or the myometrium region [15,24]. It was reported previously, when stromal cells were seeded at clonal density (low cell density), 1.25% of cells formed individual colonies having more than 50 cells/colony within two weeks of plating [8]. During later passages, the initial heterogeneous population of cells becomes more homogeneous and express more MSC-specific cell surface markers [25].

Cells during culture displayed fibroblast-like morphology, and were positive for MSC-specific surface markers such as, CD29, CD44, CD90, CD105, CD140b, and CD144. Our findings are consistent with previous reports from several human and porcine studies [7,23]. However, CD9 epithelial cell surface marker was found to be negative, confirming that the cells were stromal in origin, and hematopoietic stem cell markers CD34 and CD45 were also negative, indicating their mesenchymal origin. These observations are in accordance with earlier reports [25,26]. At present there are no specific markers to identify or enrich EMSCs from endometrium [27].

Stem cell marker or pluripotent marker expression were reported in various adult stem cells, human bone marrow and endometrium [28,29], and various porcine tissues such as umbilical cord matrix cells, skin, adipose, and others [30,31,32]. In the present study we are reporting positive expression of OCT4, SOX2 and NANOG in stromal derived EMSCs at passage 3 both at the protein and gene level by employing Western blotting, immunostaining and PCR amplification method, however in porcine skin fibroblast cells these markers were absent. Adult porcine fibroblast did not express *OCT4* pluripotent marker [33,34] proving that pluripotent gene expressions are confined to adult stem cells. However, Miernik and Karasinski [7] failed to report the expression of *OCT4* and other pluripotent genes in porcine EMSCs, this contrasting result might be due to the presence of heterogeneous cells, differences in cell isolation, passages, and experimental methods. Pluripotent markers including OCT4, which were expressed endogenously inside cells, could not be employed for isolating endometrial MSCs [27]. Further the positive expressions of these pluripotent markers are not determining factors to prove their potency in somatic tissue cells [30].

Multi-lineage differentiation is a functional property of MSCs in which undifferentiated self-renewal cells were differentiated to specialized adult cells by expressing phenotypic differentiation markers and transcription factors [27]. Earlier it was reported that porcine MSCs are capable of being differentiated into fat, bone and cartilage upon exposure to specific induction media [2,3,14,35]. Following induction into adipogenic and osteogenic lineages, EMSCs exhibited oil droplets and mineralized matrix containing calcium nodules, similar to earlier findings in porcine MSCs [3,36]. Adipogenesis is a two-step process, wherein during the first step preadipocytes are formed while retaining fibroblast morphology, however at the final maturation step lipid vacuoles are formed inside the whole adipocytes [9]. The expression of adipogenic lineage genes, *FABP* and *LPL*, a late marker of adipogenesis, and *AP2* which triggers preadipocytes to adipocyte differentiation, were highly expressed in differentiated cells, indicating that EMSCs have potential for adipogenesis. Similarly osteogenic lineage genes, *ON* for initiation of mineralization, *RUNX2* for osteoblast differentiation, and *BG* for mineralization of bone, were also increasingly expressed in differentiated cells. This positive gene expression pattern indicates that MSCs can efficiently respond to signaling molecules, and differentiate to multilineages, proving their plasticity [9]. These findings are consistent with previously published reports on porcine-derived MSC differentiation to multilineages [37]. Further, a study on human endometrial stem cells has reported similar findings related to differentiation ability of mesenchymal lineages [38]. To our knowledge this is the first report on lineage-specific gene expression pattern in porcine stromal-derived EMSC.

Transdifferentiation of MSCs can be described as transformation of cells from one tissue lineage to a distinct lineage with acquisition of new markers and function of that distinct cell type. The nuclear reprogramming and changes in key developmental genes are mediated by trans-differentiation and these processes arise naturally during *in vivo* tissue damage [39]. Plasticity of adult stem cells are well demonstrated in bone marrow MSCs where they migrate via the blood stream to damaged tissues like heart, muscle, liver, nervous system, pancreas and skin to transdifferentiate into cardiomyocytes, skeletal muscle cells, hepatocytes, neurons, β-cells and keratinocytes respectively [40,41,42,43]. In pig, umbilical cord matrix, bone marrow and adipose derived MSCs were reported to possess transdifferentiation capacity [1,5,44].

Similar to human MSCs, porcine MSCs (pMSCs) are capable of transdifferentiating into ectodermal lineages [9]. Previously, porcine Wharton’s jelly derived MSCs were successfully differentiated into neuron-like cells *in vitro* and displayed morphological, biochemical characteristics along with neuron specific marker expression [1]. Upon induction using an RA protocol, EMSCs transdifferentiated into neuron-like cells by expressing neuronal specific markers and genes analyzed by immunocytochemistry and RT-qPCR. Our results are consistent with studies conducted using human and porcine MSCs derived from various tissues [5,22,45]. Previously BM-MSCs derived from porcine bone marrow extract, differentiated into neuron-like cells upon induction with RA; they displayed neuronal phenotype with the expression of neuron-specific markers [5]. It was reported that chemical compounds such as BHA, BME and DMSO which were widely used for neuronal induction are known to induce stress on cells to shrink the cytoplasm and stimulate neuronal morphology, and these induced cells also fail to exhibit electrophysiology properties, a distinct character of neuronal cells [5,18,21].

Though the neuronal differentiated MSCs displayed morphology and gene expression patterns similar to neurons, they should possess electrophysiological properties of a functional neuron [21]. The neurons are electrically active at rest (resting potential) due to the presence of voltage difference by movement of ions (K^+^–Na^+^) between inside and outside of the cell. We further confirmed the electrophysiological potential of transdifferentiated porcine EMSCs into neuron-like cells. Whole cell patch clamp is a most commonly employed technique to study electrophysiological properties of neurons, during which strong suction is applied to disrupt the cell membrane and create access to intracellular spaces. This technique can be used to record both ionic current and changes in membrane potential by applying either voltage clamp mode or current clamp mode respectively. The presence of the K^+^ and Na^+^ currents, which contribute to generation of action potential, was investigated by applying the protocols given in the Methods section using K^+^- and Cs^+^-based pipette solutions, respectively.

The neuronal DEMSCs elicited large K^+^ outward currents compared to control EMSCs and Na^+^ currents were only recorded in neuronal differentiated cells, but not in control, although their amplitudes were very small. Both immature neurons and non-neurons are unable to fire spontaneously or evoke action potential, however immature neurons possess active K^+^ and Na^+^ ion voltage-gated channels [21]. Our results showed that voltage dependent K^+^ and Na^+^ channels were functional and actively expressed in neuronal differentiated EMSCs. There are different types of voltage-gated channels expressed in neurons but for generation of action potential the existence of the Na^+^ voltage-gated channel is considered critical [46]. Nerve growth factors are known to play an important role in a variety of neuronal differentiation patterns, and are also responsible for increased expression of Na^+^ voltage-gated channel, as reported earlier [47].

Although human adipose-derived MSCs, human endometrial derived MSCs were previously known to exhibit electrophysiological properties [11,45], there was no report from porcine neuronal differentiated EMSCs. We presume that our protocol may not be efficient in producing mature neurons but generate immature neurons with active ion channels as opposed to chemical inducers, which fail to exhibit these intrinsic properties of neurons upon induction. There is a greater challenge for researchers to obtain completely differentiated and functional neurons from MSCs to treat neurodegenerative disorders. Also due to shortage of organs for transplantation in humans, xenotransplantation is gaining attention, and pig, being similar to humans in organ morphology, will be carefully evaluated for suitability.

## 4. Materials and Method

All animal samples were collected and handled following the approval of the Research Ethical Committee of Gyeongsang National University, animal center for biomedical experimentation under set guidelines (GNU-140305-P0016, 5 March 2014).

### 4.1. Chemicals and Media

All chemicals used in this study were purchased from Sigma Chemicals Company (Sigma, St. Louis, MO, USA) and media, serum and buffers from Gibco (Invitrogen, Burlington, ON, Canada) unless otherwise specified.

### 4.2. Isolation and Culture of Porcine Endometrial Stromal Cell, Bone Marrow Cells and Ear Skin Fibroblast

Porcine (*Sus scrofa*) uterus with ovaries (*n* = 6) from non-pregnant six-month-old female were obtained from the local abattoir and transported to the laboratory on ice-cold Dulbecco’s phosphate buffer saline (DPBS) within 1 h of collection. The uteri were cut open, washed twice with sterile Ca^2+^ and Mg^2+^ free DPBS (pH 7.4), followed by removal of endometrial and myometrial tissues with scissors. The stromal layer was finely minced into fragments followed by digestion in DPBS supplemented with 0.1% collagenase type II and 40 μg/mL deoxyribonuclease type I in a 37 °C incubator with vigorous pipetting at every 15 min interval for 1 h. After digestion, the cell suspension was filtered through a 100 and 40 μm cell strainer (BD Falcon, Bedford, MA, USA), further digestion was prevented by adding Advanced Dulbecco’s modified eagle’s media (ADMEM) containing 10% fetal bovine serum (FBS) and washed 2 times in ADMEM containing 10% FBS by centrifugation at 100× *g* for 5 min. Stromal cells were seeded at clonal density of 500 cells/cm^2^ in ADMEM supplemented with 10% FBS, 100 U/mL penicillin and 100 μg/mL streptomycin in 10 cm^2^ culture dish, and incubated at 38.5 °C in a humidified atmosphere at 5% CO_2_ in air. Once the colonies appeared after 48 h, only large and densely packed, fast growing colonies were picked using a cloning cylinder (C1059, Sigma Aldrich) and cultured into a new dish. Upon confluence, cells were dissociated with 0.25% trypsin-EDTA solution and sub-cultured into 1:4 ratios till passage 3. All further experiments were carried out using cells from passage 3.

Bone marrow mesenchymal stem cells (BM-MSCs) were extracted from porcine bone marrow [5], and fibroblasts (FB) were isolated from porcine ear skin tissue [48] as previously reported and passage 3 cells were used as positive and negative control for future experiments.

### 4.3. CD Markers Analysis by Flow Cytometry and Polymerase Chain Reaction

EMSCs were characterized for presence for mesenchymal markers (CD29, CD44, and CD90), absence for epithelial cell marker CD9 and hematopoietic marker CD34 and CD45 using flow cytometry (BD FACSCalibur, Becton Dickinson, San Jose, CA, USA) using a previously published protocol [5]. Briefly cultured stromal cells at ~90% confluence were fixed with 3.7% formaldehyde for 1 h, followed by incubation with fluorescein isothiocyanate (FITC) conjugated mouse Anti-CD9, CD34 CD44, CD45 and CD90. For evaluation of CD29 expression, cells were incubated in primary antibody (mouse anti-pig, 5 µg/mL, BD Pharmingen™, Becton Dickinson) for 1 h at 4 °C. The cells were washed in DPBS and incubated with secondary antibody consisting of FITC-conjugated goat anti-mouse IgG (5 µg/mL, BD Pharmingen™) for 1 h at 4 °C. The standard was established by isotype match control. A total of 10,000 FITC-labeled cells were measured using BD FACSCalibur with Cell Quest software.

CD markers (*CD105*, *CD140b*, and *CD144*) were analyzed by polymerase chain reaction (PCR). Briefly total RNA from passage 3 cells were extracted and reverse transcription of purified total RNA (2 μg) was performed using Omniscript reverse transcription kit (Qiagen, Hilden, Germany) with random hexamer primers (Invitrogen, USA) in a 20 μL reaction mixture at 37 °C for 1 h, followed by PCR amplification using Maxime PCR Premix (iNtRON Biotechnology, Seongnam, Korea) with specific primers shown in Table 1 under the following conditions; pre-denaturation at 94 °C 10 min, denaturation at 94 °C for 30 s, annealing at 56–64 °C for 30 s, elongation at 72 °C for 45 s and final extension at 72 °C for 10 min for 35 cycles using a Eppendorf Master cycler (Eppendorf, Hamburg, Germany). Hydroxymethylbilane synthase (*HMBS*) was used as an internal control gene. PCR products were loaded on 1.5% agarose gel containing 1 µg/mL red safe stain™ (iNtRON Biotechnology) for electrophoresis.

**Table 1:** RT-PCR and RT-qPCR primers sequences specific to porcine MSCs and differentiated cells.

Gene	Primer Sequence (5'–3')	Products Sizes (bp)	Annealing Tm (°C)	Reference/Accession Number
*OCT4*	F-AGGTGTTCAGCCAAACGACC	335	60	Carlin *et al.*, 2006
	R-TGATCGTTTGCCCTTCTGGC			
*SOX2*	F-GCCTGGGCGCCGAGTGGA	443	64	Carlin *et al.*, 2006
	R-GGGCGAGCCGTTCATGTAGGTCTG			
*NANOG*	F-ATCCAGCTTGTCCCCAAAG	438	60	Carlin *et al.*, 2006
	R-ATTTCATTCGCTGGTTCTGG			
*CD105*	F-CGCTTCAGCTTCCTCCTCCG	281	56	Miernik & Karasinski, 2012
	R-CACCACGGGCTCCCGCTTG			
*CD140b*	F-TACGTGCCCATGCTGGACATG	175	54	Miernik & Karasinski, 2012
	R-TGGTAGCTGAAGCCCACGAG			
*CD144*	F-TGCAACGAGCGGGGCGAGTT	220	56	Miernik & Karasinski, 2012
	R-CGCCGCCCTCCTCATCGTA			
*FABP*	F-TGGTACAGGTGCAGAAGTGGGA	100	60	NM_001002817
	R-GCCGTGACACCTTTCATGATACA			
*LPL*	F-CAAACTTGTGGCTGCCCTAT	202	60	Kumar *et al.*, 2007
	R-AAGGCTGTATCCCAGGAGGT			
*AP2*	F-AACCCAACCTGATCACTG	192	60	AF102872.1
	R-TCTTTCCATCCCACTTCTGC			
*ON*	F-TCCGGATCTTTCCTTTGGTTTCTA	187	60	Kumar *et al.*, 2007
	R-CCTTCACATCGTGGCAAGAGTTTG			
*RUNX2*	F-CAGACCAGCAGCACTCCATA	167	60	XM_003482203
	R-AACGCCATCATTCTGGTTAG			
*BG*	F-GTCGTCCAGTGCTCTGACCT	93	60	XM_003135475
	R-GGAGCTCGGAGATGTCGTTA			
*B3T*	F-CAGAGCAAGAACAGCAGCAGCTACTT	227	60	XM_003480812
	R-GTGAACTCCATCTCATCCATGCCCTC			
*NFM*	F-GTCAGACCAGGCAGAAGAGG	222	60	Kumar *et al.*, 2012
	R-GATTTGGGCATAGGGGATTT			
*NES*	F-AGGAACCAAAAGAGGCAGGT	229	60	Kumar *et al.*, 2012
	R-TTGGGACCAGGGACTGTTAG			
*NGF*	F-CACACCGAGAGCAATGTCCC	130	60	XM_005674272
	R-CCACCCTGGCGGCTATCGCC			
*MBP*	F-GAGATGGCTCAACTCAGAACG	125	60	NM_001001546
	R-GGTTAGTATTTGCCGTGAGCA			
*MAP2*	F-GCCATTATTCGTACACCTCCA	291	60	XM_005672149
	R-AGAGCCGCATTTGGATGTCAC			
*HMBS*	F-TTCATTCCCTCAAGGACCTG	101	60	NM_001097412
	R-GGGGTGAAAGACAACAGCAT			

### 4.4. Analysis of Pluripotent Markers by PCR and Western Blotting

The expression of OCT4, SOX2 and NANOG gene and proteins were evaluated by PCR as previously mentioned in CD markers analysis using HMBS as internal control and by western blot analysis, respectively. For Western blot analysis, total protein was extracted with RIPA buffer (PIERCE, Rockford, IL, USA) containing protease inhibitor and quantified using BCA protein assay kit (PIERCE). The cell homogenates both EMSCs and porcine skin fibroblasts (20 μg) were separated on SDS-PAGE for 3 h at 100 V and wet transferred electrically onto a PVDF membrane (pre-wetted with methanol) (Biorad, Hercules, CA, USA) for 2 h at 200 V. After being blocked with 5% bovine serum albumin (BSA) in Tris-buffered saline (1X-TBS) (200 mM Tris (pH 7.5), 500 mM NaCl) for 1 h at room temperature (rt) followed by a wash in TBST-0.1% (Tween 20), the PVDF membrane was incubated with 1:200 dilution of OCT4, SOX2 and NANOG (Santa Cruz Biotechnology, Santa Cruz, CA, USA) and 1:1000 dilution of GAPDH (Cell Signaling Technology, Boston, MA, USA) antibody in 0.1% TBST-5% BSA overnight at 4 °C. After washing 3 times in 0.1% TBST, the membrane was incubated with a 1:3000 dilution of goat conjugate anti-rabbit IgG-HRP or donkey conjugate anti-goat IgG-HRP (Santa Cruz Biotechnology) in 0.1% TBST-5% BSA for 1 h at rt. Immunoreactivity was detected by enhanced chemiluminescence (ECL; Supersignal^®^ West Pico Chemiluminescent substrate, PIERCE), and then exposed to X-ray film (FUJI Photo Film Co., Ltd., Tokyo, Japan). Data are representative of two experiments from independent cell cultures.

### 4.5. Differentiation into Adipocytes and Osteocytes

EMSCs at passage 3 with ~80% confluence were analyzed for *in vitro* differentiation into adipocytes using previously published protocol with minor changes [49]. Adipogenic differentiation was conducted in ADMEM supplemented with 10 μM insulin, 100 μM indomethacin and 1 μM dexamethasone for 21 days by changing fresh medium every 3 days. The lipid droplets were detected by Oil Red O staining.

Osteogenesis was induced in ADMEM supplemented with 10 mM sodium β-glycerophosphate, 0.05 mM ascorbic acid and 1 μM dexamethasone [30]. After 21 days of induction, the mineralization and calcium deposition was detected by Von Kossa and Alizarin Red staining, respectively. Cells maintained in ADMEM supplemented with 10% FBS were used as undifferentiated control.

### 4.6. Neurogenic Differentiation

Neurogenesis of EMSCs at 80% confluence was induced in ADMEM supplemented with 10% FBS under the Geltex LDEV-free coated plates (Gibco, Grand Island, NY, USA) according to previously described published protocols [19] with minor modification. Before neurogenic induction, cells were preinduced for 24 h in ADMEM supplemented with 10% FBS, 10 ng/mL basic fibroblast growth factor (bFGF) and 20 ng/mL epidermal growth factors (EGF), followed by induction in ADMEM with 30 μM trans-retinoic acid (RA) for 15 days by replacing fresh medium containing RA every 72 h.

### 4.7. RNA Isolation, cDNA Synthesis and Real Time Quantitaive PCR (RT-qPCR) Analysis

Total RNA from EMSCs at passage 3 and differentiated EMSCs into adipogenic, osteogenic and neurogenic like cells were isolated using an RNeasy mini kit (Qiagen) and quantified using UV BIO Spectrophotometer OPTIZEN 3220 (Mecasys Co., Ltd., Daejeon, Korea), and quality was assessed using RNA gel. Reverse transcription of purified total RNA (2 μg) was performed using Omniscript reverse transcription kit (Qiagen) with random hexamer primer (Invitrogen) in a 20 μL reaction mixture at 37 °C for 1 h.

Real time quantitative PCR (RT-qPCR) analysis was performed using a Rotor Gene Q (Qiagen) machine with 50 ng cDNA quantified with Rotor-Gene™ 2X SYBR^®^ Green mix (Qiagen) supplemented with 1 µM forward and reverse primers shown in Table 1. The RT-qPCR protocol consisted of pre-denaturation at 95 °C for 10 min; 40 PCR cycles at 95 °C for 10 s, 60 °C for 6 s and 72 °C for 6 s; melting curve from 60 to 95 °C by 1 °C per second; cooling at 40 °C for 30 s according to manufacturer’s protocol with minor modification. Rotor-Gene Q Series Software (Qiagen) was used to determine melting curves, amplification curves, and cycle threshold values (*C*_t_ values). The expression levels of genes were normalized against the corresponding HMBS expression. All products in triplicate run were confirmed by agarose gel electrophoresis for nonspecific amplification with negative control.

### 4.8. Immunofluorescence Staining

The pluripotent markers from passage 3 EMSCs and neuronal specific markers from both induced and control EMSCs were analyzed by immunofluorescence staining as in a previously described protocol [5]. Briefly the cells were washed with DPBS, fixed with 3.7% formaldehyde for 50 min and permeabilized by 0.2% Triton X-100 supplemented with 2% BSA. After blocking with 2% BSA in DBPS for 1 h, the cells were reacted with primary antibodies (1:100), pluripotent markers-OCT4-goat polyclonal, SOX2-rabbit polyclonal, and neuronal markers-neurofilament M (NFM)-goat polyclonal, β-tubulin III (B3T)-mouse monoclonal, nerve growth factor (NGF)-rabbit polyclonal, tropomyosin receptor kinase-A (TRKA)-goat polyclonal (Santa Cruz Biotechnology) at 4 °C for 1 h. After incubation, cells were washed three times with DPBS, followed by incubation with FITC conjugated secondary antibodies (1:100) for 1 h. Cells were then counterstained with 1 µg/mL 4',6-diamidino-2-phenylindole (DAPI) for 5 min at rt. After being mounted with Vectashield^®^ (Vector Laboratories, Inc., Burlingame, CA, USA), cells were observed under fluorescence microscope (Leica, Wetzlar, Germany).

### 4.9. Electrophysiological Study

For electrophysiological recording, both control and neuronal differentiated cells were cultured on the poly-l-lysine-coated glass coverslips. Whole-cell patch-clamp technique using a patch clamp amplifier (Axopatch 200, Axon Instruments, Union City, CA, USA) was performed at rt to record K^+^ and Na^+^ currents. The bath solution composed of 135 mM NaCl, 5 mM KCl, 1 mM CaCl_2_, 1 mM MgCl_2_, 10 mM glucose and 10 mM HEPES and adjusted its pH to 7.4. The pipette resistance ranged between 2–4 MΩ when filled with pipette solution (pH 7.3) consisting 150 mM KCl, 1 mM MgCl_2_, 5 mM EGTA, and 10 mM HEPES for K^+^ currents and 140 mM CsCl, 2 mM KATP, 4 mM MgCl_2_, 10 mM EGTA, 10 mM HEPES and 1 mM tetraethylammonium (TEA) for Na^+^ currents. To block K^+^ channel and to recording Na^+^ currents, TEA was added to bath solution. Na^+^ and K^+^ currents in both control and differentiated cells were evoked by voltage steps (between −40 and +50 mV for Na^+^ currents, between −120 and +60 mV for K^+^ currents) from a holding potential of −80 mV. To block sodium channel, lidocaine (100 μM) and bupivacaine (100 μM) were used in bath solution. The recorded signal was filtered at 2 kHz and transferred to a computer using the Digidata 1322A interface (Axon Instruments). Acquired whole-cell currents were analyzed with the pCLAMP (version 10.4, Axon Instruments) and Origin^®^ (Microcal Software, Inc., Northampton, MA, USA) programs.

### 4.10. Statistical Analysis

Differences among proportional data were analyzed by SPSS 21.0 (SPSS Inc., Chicago, IL, USA). Data was expressed as means ± SE. Comparisons of mean values among groups were performed using student *t*-test or ANOVA with Tukey’s test. Differences were considered to be significant at *p* < 0.05.

## 5. Conclusions

In summary, porcine endometrial stromal-derived mesenchymal stem cells (EMSCs) exhibited plastic adherence upon culturing, positive for mesenchymal cell surface markers, expressed pluripotent markers, differentiation ability into adipocytes and osteocytes and finally possessed transdifferentiation potential to neuron like cells with functional voltage-dependent K^+^ and Na^+^ channels evaluated by electrophysiology. Porcine EMSCs serve as good candidate in studying molecular mechanisms involved during transdifferentiation and its efficiency during *in vivo* transplantation.
